# Wear Behavior of the Multiheterostructured AZ91 Mg Alloy Prepared by ECAP and Aging

**DOI:** 10.1155/2020/4873286

**Published:** 2020-07-08

**Authors:** Bingqian Xu, Jiapeng Sun, Zhenquan Yang, Jing Han, Dan Song, Jinghua Jiang, Aibin Ma

**Affiliations:** ^1^College of Mechanics and Materials, Hohai University, Nanjing 210098, China; ^2^School of Mechanical and Electrical Engineering, China University of Mining and Technology, Xuzhou, 221116 Jiangsu Province, China

## Abstract

The microstructure design based on the development of heterostructure provides a new way for high strength and ductility Mg alloys. However, the wear property, as an important service performance, of Mg alloys with heterostructure is scarcely investigated. In this work, a high strength and ductility AZ91 Mg alloy with multiheterostructure was prepared *via* a processing route combined industrial-scale equal channel angular pressing (ECAP) and aging. The multiheterostructure consists of the heterogeneous grain structure and heterogeneous precipitates. The dry sliding wear behavior of this multiheterostructured (MH) alloy is investigated compared to the as-cast alloy. The impacts of the applied load and duration time on the wear volume and coefficient of friction (COF) are analyzed, and the wear mechanism is further discussed. The result indicates that although the MH alloy exhibits high-desirable strength-ductility synergy, it shows a poorer wear resistance but a relatively lower COF compared to the as-cast alloy at the present condition. The wear mechanism of both alloys mainly involves abrasive wear, as well as mild adhesion, delamination, and oxidation. In comparison, the MH alloy shows relatively severe adhesion, delamination, and oxidation. The poor wear resistance of the MH alloy at the present dry sliding wear condition is linked to the abundant grain boundaries and fine precipitates. Therefore, one should reasonably use the MH Mg alloy considering the service conditions to seek advantages and avoid disadvantages.

## 1. Introduction

According to the need for lightweight of the structural materials, magnesium (Mg) alloys, as the lightest structural metallic materials, are widely applied in the automobile, aerospace, biomedicine application, and electronics industries [1–5]. Moreover, profiting from all kinds of processing methods, numerous high strength Mg alloys have been produced in the laboratory and even some Mg alloys have been commercialized, such as Mg-Al-Zn (AZ)-typed alloys and Mg-Zn-Zr (ZK)-typed alloys [6–9]. However, the absolute strength and ductility of Mg alloys at room temperature are still much lower than that of Al alloys, Ti alloys, and steels, which restrict their widespread commercial applications. Therefore, great effort has been made to enhance the mechanical properties of the Mg alloys in the last few decades [10–13].

Due to the well-known Hall-Petch law, the ultrafine/nano-grained Mg alloys are intensely pursued to address the issues of poor strength and ductility [14–19]. In recent years, the microstructure design based on the development of the heterostructure provides a new way for the high-performance materials [20–23]. Many studies indicate that the heterostructure brings into much higher strength and ductility in several Mg alloys than the generally developed ultrafine grain structure [24, 25]. For example, Ramezani et al. [26] developed a GWZ Mg-8.1Gd-4.3Y-1.6Zn-0.4Zr (wt. %) alloy with fine-grained bimodal microstructures *via* multiaxial forging. The forged alloy exhibited an excellent ultimate tensile strength (UTS) and ductility of 581 MPa and 15.9%, respectively. Jiang et al. [27] processed a heterogeneous Mg–1Gd/Mg–13Gd alloy laminate *via* accumulative extrusion bonding (AEB), whose strength and ductility were much superior to that of the individual component material. Wu et al. [28] demonstrated that the bimodal structure brought into high strength in the Mg-15Gd-1Zn-0.4Zr (wt. %) alloys. Xu et al. [29] prepared a bimodal-structured Mg-8.2Gd-3.8Y-1Zn-0.4Zr (wt. %) alloy using hot extrusion and aging, which had a superior strength–ductility balance. In our previous work, the laboratory scale and industrial-scale multiheterostructured AZ91 alloys were prepared *via* the combined processing route of ECAP and aging [30–32]. The multiheterostructured AZ91 alloy exceeded the known strength and ductility limits of the AZ91 alloy.

Compared to the abundant investigation on the mechanical properties, the wear behavior of Mg alloys is scarcely investigated, so that we still cannot confirm whether these high-performance Mg alloys with heterostructure have excellent wear resistance. The present work aims to investigate the dry sliding wear behavior of a multiheterostructured AZ91 alloy produced by a processing route combined industrial-scale ECAP with aging. The impacts of the applied load and duration time on the wear volume and coefficient of friction (COF) were investigated, and the wear mechanism was further discussed.

## 2. Experimental Procedure

A commercial as-cast AZ91 alloy ingot with a nominal chemical composition of 9 wt. % Al, 1 wt. % Zn, and 0.5 wt. % Mn was used in this work. A combined processing route of industrial-scale ECAP with aging was used to prepare the multiheterostructure AZ91 alloy. The received AZ91 alloy was referred to as “AC alloy.” The AC alloy was firstly homogenized at 420°C for 24 h followed by water quenching. Next, a homemade upscaled rotary die equal channel angular pressing (RD-ECAP) with four equal square channels (50 mm × 50 mm × 100 mm) was continually performed for 16 passes with a ram speed of 3.5 mm/s at a constant temperature of 350°C, followed by rapidly water cooling. Finally, the ECAPed samples were isothermally aged at 200°C for 15 h, where the peak hardness was obtained. More details on the processing route can be found in our previous work [30], and the resultant sample was designated as “MH alloy.”

**Figure 1:**
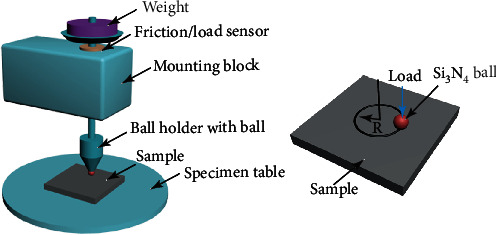
Schematic illustration of the ball-on-disc facility for the wear test.

The wear test was performed by a ball-on-disc facility (MFT-3001 type-Lanzhou Huahui Instrument Technology Co., LTD., China), as illustrated in Figure 1. Before wear testing, the samples were ground to 800 grit SiC sandpapers and cleaned with ethanol. The roughness of the samples is *Ra* 0.31 *μ*m. The Si_3_N_4_ ball with a diameter of 6 mm was used. All the tests were performed at a velocity of 300 r/min with a revolution radius of 2.5 cm. Since duration time (*T*) and applied load (*L*) have a great influence on wear behavior, different duration times (*T* = 10 min, 30 min, and 90 min) and applied loads (*L* = 4.9 N, 9.8 N, and 14.7 N) were chosen to reveal their effect on the wear behavior of the AC and MH alloys. When the duration time was varied, the constant applied load was 4.9 N. When the applied load was varied, the constant duration time was 30 min. For each state, at least two specimens were tested. After wear testing, the samples were ultrasonically cleaned with ethanol for 5-10 min and then stored in kerosene. The wear track was characterized by the surface profile meter (TIME3222 Beijing Time High Technology LTD, China). The wear volume was calculated according to the measured cross-section profiles, which was expressed as the product of the cross-sectional area and the length of the wear track. The micro-hardness of the samples was measured by an HXD-1000TC microhardness-testing instrument with a load of 4.9 N and a dwelling time of 15 s.

The microstructures were characterized by scanning electron microscopy with the secondary electron detector (SEM) equipped with an energy dispersive spectrometer (EDS) and electron backscattered diffraction (EBSD). For SEM observation, the samples were ground to 2000 grit SiC sandpaper successively, mechanically polished by a 1.5 *μ*m diamond suspension, and then etched by a mixture solution of acetic acid, picric acid, ethanol, and distilled water. Whereas, the EBSD samples were ground, polished, and then ion milled.

## 3. Results and Discussions

### 3.1. Microstructure and Mechanical Properties

**Figure 2:**
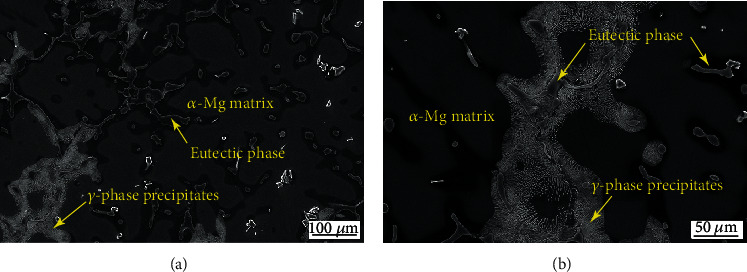
SEM micrographs of the AC alloy. The low-magnification (a) and high-magnification (b) images.

Figure 2 shows the SEM micrographs of the AC alloy. This alloy is featured with a typical dendritic structure which consists of a coarse *α*-Mg matrix with an average grain size of 180 *μ*m, *γ*-phase precipitates (Mg_17_Al_12_), and interdendritic eutectic phase.

**Figure 3:**
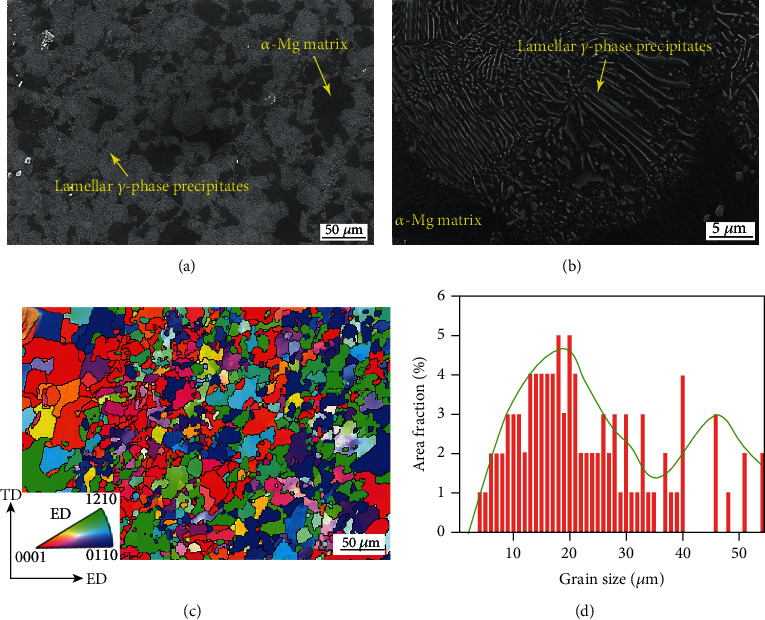
SEM micrographs (a, b), EBSD inverse pole figure mapping (c), and grain size statistics (d) of the MH alloy.

The present used combined processing route brings into a multiheterostructured (MH) AZ91 alloy, which was investigated in detail in our previous work [30]. The microstructure of the MH alloy is characterized by a heterogeneous grain structure and a heterogeneous precipitate structure, as shown in Figure 3. The SEM indicates that the fine lamellar *γ*-phase precipitates cover most of the grains, but some grains are still precipitate-sparse/free, indicating a heterogeneous precipitate structure, as shown in Figures 3(a) and 3(b). Here, the lamellar precipitates are formed during aging in a discontinuous mode. Besides, some cobblestone-like *γ*-phase particles can be found on the grain boundaries, which are dynamically precipitated during ECAP processing. The EBSD observation shows that the heterogeneous grain structure is comprised of coarse grains with an average size of 44.2 *μ*m and fine grains with an average size of 18.7 *μ*m, as artificial critical grain size of 35 *μ*m is chosen, as shown in Figures 3(c) and 3(d). The volume fraction of the fine grains reaches 84.8%.

**Figure 4:**
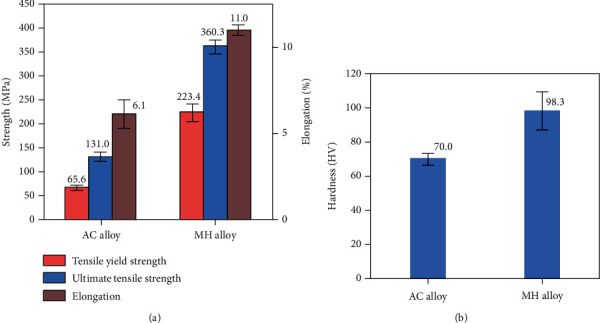
The mechanical properties [7, 30] (a) and microhardness (b) of the AC and MH alloys.

The AC alloy has poor mechanical properties, as shown in Figure 4(a). The multiheterostructure brings a superior combination of high strength and good ductility to the AZ91 alloy. The tensile yield strength (TYS), ultimate tensile strength (UTS), and elongation (EL) of the MH alloy are improved by 240.5%, 175.0%, and 80.3%, respectively, compared to the AC alloy (Figure 4(a)). Moreover, the microhardness increment of the MH alloy is over 40%, as shown in Figure 4(b).

### 3.2. Wear Behavior

**Figure 5:**
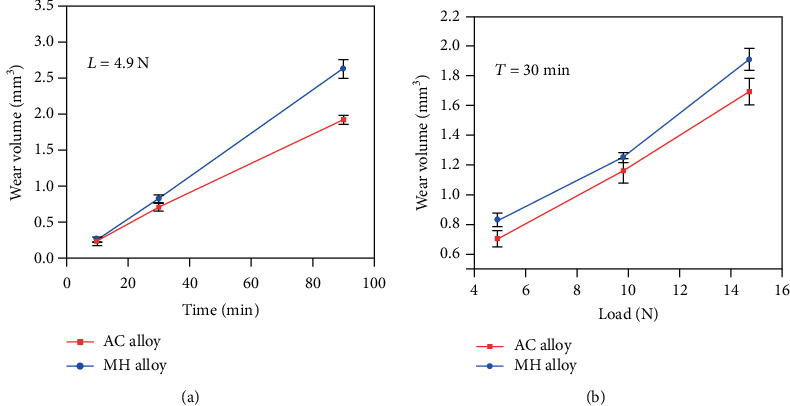
Wear volume of the AC and MH alloys as the function of the duration time *T* (a) and the applied load *L* (b).

Figure 5(a) shows the time evolution of the wear volume. The wear volume almost linearly increases with increasing duration time both for the AC alloy (from 0.234 mm^3^ to 1.915 mm^3^) and the MH alloy (from 0.247 mm^3^ to 2.622 mm^3^). The MH alloy exhibits a more rapidly increasing trend of the wear volume than the AC alloy, indicating a larger wear rate. Therefore, the MH alloy has a larger wear volume than the AC alloy under all duration time, although the difference is hard to be perceived under a short duration time.  Figure 5(b) presents the wear volume as a function of the applied load. The wear volume of both alloys increases gradually with increasing the applied load and its growth trend of both alloys is similar. Apparently, the wear volume of the MH alloy (from 0.826 mm^3^ to 1.908 mm^3^) is larger than that of the AC alloy (from 0.701 mm^3^ to 1.690 mm^3^) at all the applied loads. The present results demonstrate that the MH alloy exhibits a poorer wear resistance than the AC alloy, although it has better mechanical properties.

**Figure 6:**
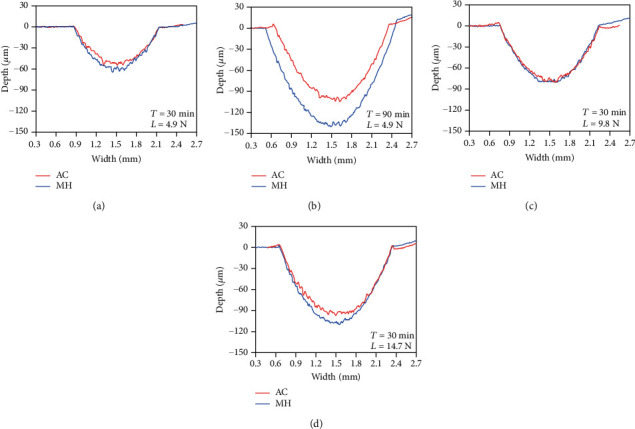
The wear track cross-section profiles of the AC and MH alloys under the applied load *L* of 4.9 N (a, b) and the duration time *T* of 30 min (c, d).

Figure 6 shows the wear track cross-section profiles of the AC and MH alloys. Under short duration time or small applied load, the wear track of two alloys is similar, while it is slightly deeper for the MH alloy, as shown in Figures 6(a) and 6(c). Increasing duration time, both the wear track depth and width of the MH alloy are growing faster than that of the AC alloy, as shown in Figure 6(b), indicating rapidly increased wear volume. Increasing the applied load, the discrepancy of the wear track depth between the AC alloy and the MH alloy dramatically grows, but the wear track widths of two alloys are similar.

**Figure 7:**
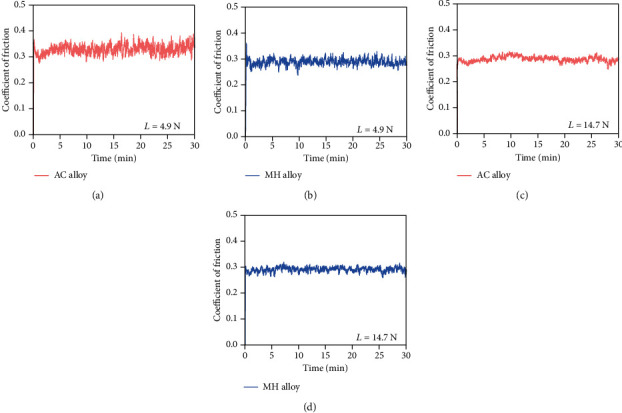
The fluctuation of the COF values of the AC (a, c) and MH (b, d) alloys under the applied load *L* of 4.9 N (a, b) and 14.7 N (c, d).

Figure 7 shows the evolution of the COF for the AC and MH alloys under the applied load of 4.9 N and 14.7 N. After a rapid increase, the COF tends to be steady for all the samples, although the fluctuation is still visible. It is evident that the AC alloy shows more drastic COF fluctuation than the MH alloy. Furthermore, the COF fluctuation is influenced by the applied load, as shown in Figure 7. To be specific, the lower applied load, the more fluctuant COF.

**Figure 8:**
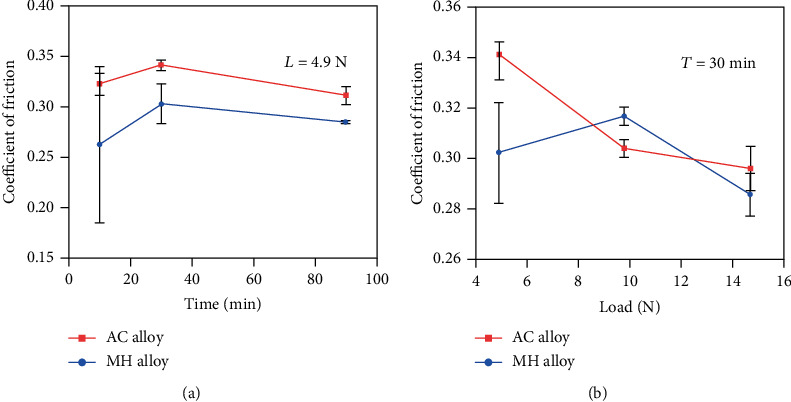
The average COF of the AC and MH alloys versus the different duration times *T* (a) and applied loads *L* (b).

The average COF of the AC and MH alloys during the steady wear stage was calculated, and the result is shown in Figure 8. As the duration time increases, the COF of these two alloys increases first and then decreases. The COF of the AC alloy is higher than that of the MH alloy under different duration times (Figure 8(a)). The highest COF is 0.341 for the AC alloy, while it is decreased to 0.302 for the MH alloy. Nevertheless, under different applied loads, the variation trend of COF of the AC and MH alloys is quite different, as shown in Figure 8(b). The COF of the AC alloy (from 0.341 to 0.296) rapidly decreases, followed by a relatively gentle descent. In contrast, the COF of the MH alloy first increases and then decreases with increasing the applied load. Therefore, the MH alloy exhibits a low COF at an applied load of 4.9 N (0.262) and 14.7 N (0.286). As a whole, the MH alloy shows the relatively lower COF compared to the AC alloy under different duration times and applied loads.

### 3.3. Wear Mechanism

**Figure 9:**
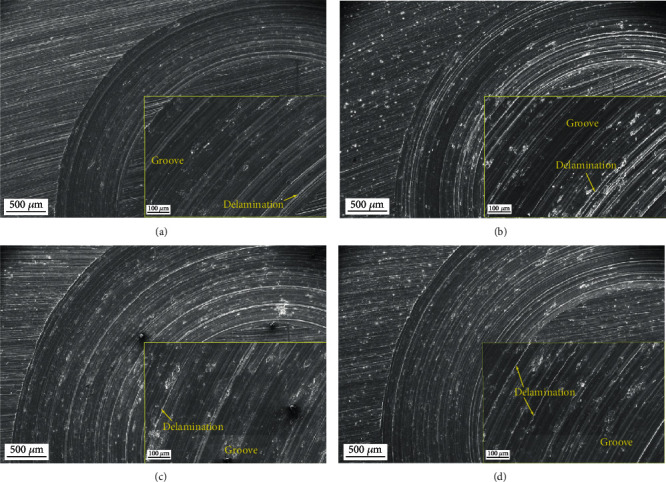
The SEM images of the typical wear surfaces of the AC alloy: (a) *T* = 10 min, *L* = 4.9 N; (b) *T* = 30 min, *L* = 4.9 N; (c) *T* = 90 min, *L* = 4.9 N; (d) *T* = 30 min, *L* = 9.8 N.

**Figure 10:**
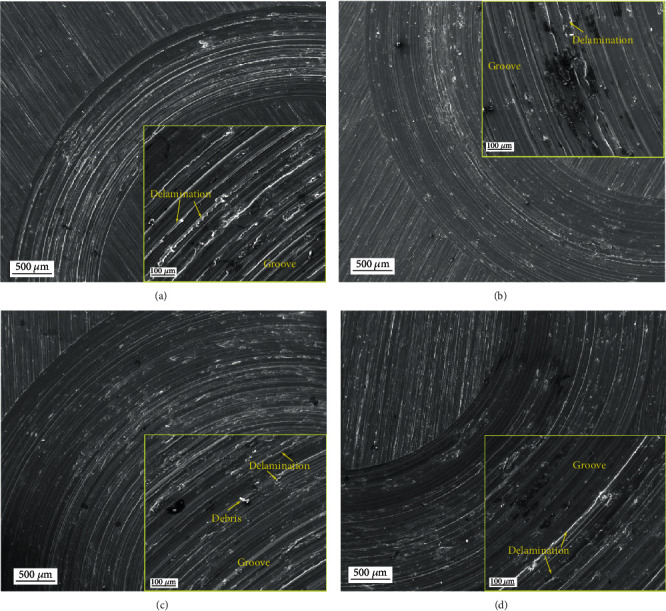
The SEM images of the typical wear surfaces of the MH alloy: (a) *T* = 10 min, *L* = 4.9 N; (b) *T* = 30 min, *L* = 4.9 N; (c) *T* = 90 min, *L* = 4.9 N; (d) *T* = 30 min, *L* = 9.8 N.

**Figure 11:**
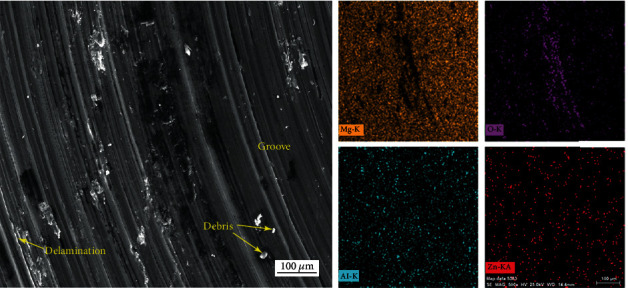
Large-magnification SEM images and EDS elemental mappings of the typical worn surfaces of the MH alloy under the applied load *L* of 4.9 N and the duration time *T* of 30 min.

The morphologies of the wear surface were further characterized using SEM combined with EDS to reveal wear mechanism. Figures 9 and 10 present the SEM images of the typical wear surfaces of the AC and MH alloys. Along the sliding direction, numerous grooves, ridges, and debris can be observed on the wear surface of both alloys at all conditions, representing the typical morphologies of abrasive wear. Therefore, the abrasive wear is the primary wear mechanism for both alloys. Besides, some adhesion marks and delamination are visible for both alloys, indicating the occurrence of adhesion wear and delamination wear. Some black stains can be found in both alloys. The EDS analysis demonstrates that these stains contain high-concentration O element, giving rise to the occurrence of oxidation wear, as shown in Figure 11. In comparison, the MH alloy shows relatively severer adhesion, delamination, and oxidation. As the duration time and applied load increase, the wear track width of both alloys increase, but it is hard to tell the difference in the wear mechanism. The present result is consistent with these previous researches that the dominant wear mechanism of the AZ91 alloy at low load and sliding is abrasive wear [33, 34], and confirms that the MH alloy has almost same wear mechanism to the AC alloy.

The MH alloy exhibits finer grains compared to the AC alloy. The fine grains benefit to the improvement in strength and ductility due to the well-known Hall-Petch law. In contrast, fine grain is a sword with double blades for the wear behavior, which not only can hinder wear due to the improved hardness but also can accelerate wear due to the abundant unstable grain boundaries. During the dry sliding wear process, the high friction heat will deteriorate the instability of the grain boundaries, which is confirmed by the observed relatively severe oxidation. Also, the modification of the precipitate microstructure impacts the wear behavior. The fine lamellar *γ*-phase precipitates in the MH alloy may be obstructive to improve wear resistance, because the small precipitates are easy to be dug out when the abrasive wear prevails. Therefore, the MH alloy exhibits larger wear volume than the AC alloy during the present dry sliding wear condition, although it has significantly improved strength, ductility, and hardness. Indeed, the decreased and improved wear resistance are both frequently reported for the ECAP metals in literature. For example, Patil et al. [35] indicated that the ECAP processed samples displayed a poorer wear resistance as compared to the as-cast sample at 20 N and 30 N loads, but a decreased wear resistance at 10 N. On contrast, Xu et al. [36] reported that the wear volume of ECAP-processed AZ31 is smaller than for the as-received unprocessed alloy. Therefore, the wear resistance of the MH alloy prepared by ECAP is not only related to its physical properties but also significantly related to the wear condition. One should reasonably use the MH alloy considering the service conditions to seek advantages and avoid disadvantages.

## 4. Conclusion

In this work, the dry sliding wear behavior of the MH AZ91 alloy with multiheterostructure was investigated, which was prepared *via* the combined processing route of industrial-scale ECAP and aging. Although the MH AZ91 alloy exhibits high-desirable strength-ductility synergy due to its special multiheterostructure, it shows a poorer wear resistance compared to the AC alloy at the present condition. As a whole, the MH alloy shows the relatively lower COF compared to the AC alloy. The difference in wear volume between two alloys is hard to be perceived under a short duration time, while the MH alloy has a much larger wear volume than the AC alloy under a long duration time. The wear volume of both alloys is almost linearly increased with the increase in the applied load. The wear mechanism of both alloys mainly involves abrasive wear, as well as mild adhesion, delamination, and oxidation. In comparison, the MH alloy shows relatively severer adhesion, delamination, and oxidation. The poor wear resistance of the MH alloy at the present dry sliding wear condition is linked to the abundant grain boundaries and small and fine precipitates.

## Data Availability

The data used to support the findings of this study are available from the corresponding author upon request.
